# Escape to the future – a qualitative study of physicians’ views on the work environment, education, and support in a digital context

**DOI:** 10.1186/s12911-023-02337-7

**Published:** 2023-10-19

**Authors:** Maria Hägglund, Anna Kristensson Ekwall, Nadia Davoody, Nasim Farrokhnia

**Affiliations:** 1 Department of Women’s and Children’s Health, Uppsala University, Dag Hammarskjölds väg 14B, Uppsala, 752 37 Sweden; 2 Uppsala University Hospital, Uppsala, Sweden; 3 Health Informatics Center, Department of Learning, Informatics, Management, and Ethics, Karolinska Institutet, Stockholm, Sweden; 4 Department of Health Sciences, Lund University, Lund, Sweden; 5 Department of Clinical Science and Education, Karolinska Institutet, Södersjukhuset, Stockholm, Sweden

**Keywords:** Video consultations, Telemedicine, Work environment, eHealth

## Abstract

**Background:**

The use of remote services such as video consultations (VCs) has increased significantly in the wake of the COVID-19 pandemic. In Sweden, private healthcare providers offering VCs have grown substantially since 2016 and have been controversial. Few studies have focused on physicians’ experiences providing care in this context. Our aim was to study physicians’ experiences of VCs, focusing on the work environment, quality of care, and educational needs.

**Methods:**

Twenty-two semi-structured interviews were performed with physicians working with VCs in Sweden, and analyzed through inductive content analysis.

**Results:**

We identified five categories; flexibility, social work environment, impact on care and society, continuous learning and career development, and organizational support. Flexibility and accessibility were considered positive features of working digitally by giving physicians control over their time and workplace and increasing patients’ timely access to healthcare. Regarding collegial contact and social activities in a digital context, the majority of the participants did not experience any significant difference compared to the physical context. Access to technical support services, educational support, and collegial support in decision-making, guidance, and consultations were described as well-functioning. Satisfied patients positively impacted the work environment, and participants felt that VCs have a positive socio-economic effect. Continuity of care was considered supported, but patients did not always prioritize this. Privacy risks were considered a challenge, as were poor development of clinical skills due to the low variation of patient cases. Working for an online healthcare provider was contributing to career advancements for junior clinicians.

**Conclusions:**

Physicians appreciate the flexibility of the digital context and seem satisfied with a work environment where they have a high level of control, but few consider this a full-time career option. The pandemic year 2020 has led to a significant increase in the implementation of VCs in traditional care systems. How this affects the work environment and continuous education needs and career development remains to be seen.

**Supplementary Information:**

The online version contains supplementary material available at 10.1186/s12911-023-02337-7.

## Background

Telemedicine has a long history in Sweden, with the first projects as early as the 1920s when Sahlgrenska University Hospital in Gothenburg established sea-to-shore radio consultations to Swedish vessels around the world [1]. The first known telemedicine trial in fact took place in 1915 when ECG signals were sent across campus at Lund University [1]. Video consultations (VCs) have also been used in the northern part of Sweden where the population is low and distances to the nearest specialist hospital are long [2]. The early telemedicine implementations were however mainly focused on consultations between specialist care and primary care, or follow-up visits with patients on healthcare’s initiative [2].

In the wake of the COVID-19 pandemic, telemedicine and VCs have become a necessity in healthcare [3, 4]. OECD defines telemedicine as “the use of ICT to deliver health care (clinical services only) at a distance” [5], and divides it into three subgroups;

1. telemonitoring, or remote patient monitoring, telehomecare, i.e. use of information and communication technologies (ICTs) to monitor health status at a distance,
2. store and forward, i.e. an encounter or consult aided by the asynchronous transmission of clinical data, and
3. interactive telemedicine, or VCs, real-time teleconsultations, virtual visits, i.e. synchronous encounters or consultations at a distance using ICTs.

In this paper, we focus on the third type of telemedicine and we will use the term video consultations (VCs), which has been used extensively in the literature [4, 6, 7], to distinguish these types of telemedicine solutions from e.g. chat functions [8], and telemonitoring [9].

In 2015, a new type of telemedicine service emerged in Sweden - first-line VCs with primary care on the patient’s initiative. The introduction of private online healthcare providers in Sweden was controversial and has been criticized on several points; draining the tax-funded Swedish healthcare system by offering unnecessary care [10], poor quality due to difficulties assessing patients online, and unequal access to care where young well-educated urban citizens are prioritized over fragile older patients with complex comorbidities. So far, however, limited research has been performed regarding this novel form of care. Studies focusing on patients’ experiences indicate that patients appreciate the easy access to care and are overall satisfied with the care they receive [11], similar to other studies of VCs [12]. Elderly patients in Sweden have however expressed ambivalence towards VCs [13], which is also reflected in the annual “The Swedes and the Internet” survey which indicated that older age groups fall behind in the adoption of eHealth and VCs [14].

Digital skills have been identified as crucial for clinicians [15, 16], not least during the rapid implementation of VCs during the pandemic. In a recent qualitative study of healthcare professionals’ perceptions of digital health competence, the authors found that the participants either reported sufficient competence or perceived a lack of skills in some specific areas [17]. From a work environment perspective, a mismatch between perceived own skills and demands posed to work with new technologies can increase stress and feelings of inadequacy, and continuous education is essential to ensure that the clinical workforce is ready to adopt digital care [18].

In a UK study of VCs at three hospitals in London, VCs were popular among some patients and staff but also proved challenging to implement in “a busy and financially stretched acute hospital setting” [19]. In a Norwegian study of general practitioners’ experiences of VCs during the Covid-19 pandemic, they expressed a clear difference in usefulness depending on whether they knew the patient or not [6]. The context of online healthcare providers is however very different from VCs integrated into physical primary care, and few studies have focused on healthcare professionals’ experiences in this context. In this study, we interviewed physicians working with one of the main Swedish private digital care providers (KRY AB) about their experiences of working with VCs in a digital-only context.

### Aim

This study aimed to explore physicians’ experiences of providing care through VCs with a special focus on the work environment, quality of care, and educational needs.

## Methods

In this study, we performed qualitative interviews with 22 physicians working for a Swedish online healthcare provider regarding their experiences of the work environment and educational needs. The consolidated criteria for reporting qualitative research (COREQ) guideline [20] was used for reporting the results.

The research team consists of 4 PhDs, all women. NF is also a medical doctor and was at the time of the study employed by the online healthcare provider from where study participants were included. AE is a registered nurse and worked both clinically and as a researcher at the time of the study. All have previous experience in qualitative research.

### Study setting and participants

The online healthcare provider mainly delivers VCs with nurses, physicians, and psychologists available through a web-based and mobile/tablet platform in Sweden. The healthcare provider makes VCs possible via chat or video directly on a smartphone. Physicians have the choice to either work from home or the main office in Stockholm. The option to work from home is utilized by many, and the healthcare provider has employees all over Sweden, and abroad. All physicians working from home are provided with a laptop installed by the organization to ensure centrally controlled security and updates. In addition to the applications for the actual VCs, the physicians have access to a communication tool (Slack) that can be used for collegial support and second opinions, access to the Swedish National Patient Overview (NPO) giving access to patients’ electronic health records from across Sweden [21], and a knowledge bank of clinical guidelines for online care.

A convenience sample of volunteer participants was included in the study. Recruitment of study participants began in January 2019 when information about the study was sent out via email to all physicians working with VCs at the healthcare provider at that time. The information was sent by an administrator at the healthcare provider, referring to the researchers as responsible for the study. It was clearly stated that participation in the study was voluntary. Thirty-three physicians expressed an interest in participating, but 11 later declined to do so for various reasons, lack of time being the most prominent. Table 1 gives an overview of the 22 study participants’ characteristics.

**Table 1:** Overview of study participants

Characteristic	Number (%)
**Sex**	
Male	11 (50%)
Female	11 (50%)
**Age**	
30–39	5 (23%)
40–49	5 (23%)
50–59	4 (18%)
60–69	5 (23%)
70–79	3 (13%)
**Level of medical training**	
Specialist	15 (68%)
Resident	3 (14%)
Not a specialist or resident	4 (18%)

The majority of our study participants worked part-time (4–20 h/week) with VCs, and part-time in traditional care. A few worked exclusively with VCs, part or full-time. Of these, a few had retired and worked online from home. A few worked from abroad, either from their own homes or to complement work at a clinic in that country. Experience working with VCs ranged between 5 months and 3 years (median 1 year).

### Data collection

A preliminary semi-structured interview guide (Appendix 1) was designed by three of the authors (MH, AKE, and NF), and later tested and refined by MH, AKE, and a health informatics master student. The interview guide was structured into four main areas of inquiry; [1] education and competence, [2] experiences of the VC, [3] work environment & quality of care, and [4] attitudes towards online care. In this study, we focus on the areas of education and competence, work environment, and quality of care. In addition, some background questions were asked at the beginning about the participants’ work experience and reasons for working with VCs.

Scheduling of interviews began in February 2019, and interviews were performed between February and April 2019, that is before the pandemic increased the use of VCs dramatically. Each interview lasted between 30 min and 1 h. Three researchers were involved in performing the interviews (MH, AKE, and a health informatics master’s student), each following the same interview guide. The interviewer introduced themselves at the start of the interview, describing their interest in the topic at hand and experience of clinical work (or lack thereof). Only the interviewer and the study participant were present during the interview. As participants in the study were located across Sweden, and some in other countries, all interviews were performed online, using the tool ZOOM. All interviews were recorded and later transcribed. No field notes were taken. No repeat interviews were carried out. Data saturation was not discussed.

### Data analysis

Data analysis was performed by all four co-authors (MH, AE, ND, and NF), following an inductive content analysis approach according to Graneheim and Lundman [22]. All authors read through all interviews and then took responsibility for each coding a subset of the interviews. Frequent meetings were held where codes were discussed and compared, and sub-categories emerged. The sub-categories were organized into categories in an iterative process that lasted until consensus on the categories was reached.

No participant checking was performed.

Ethical approval for the study was granted by the Swedish Ethical Review Authority (Dnr: 2018/2318-31/5).

## Results

**Fig. 1:**
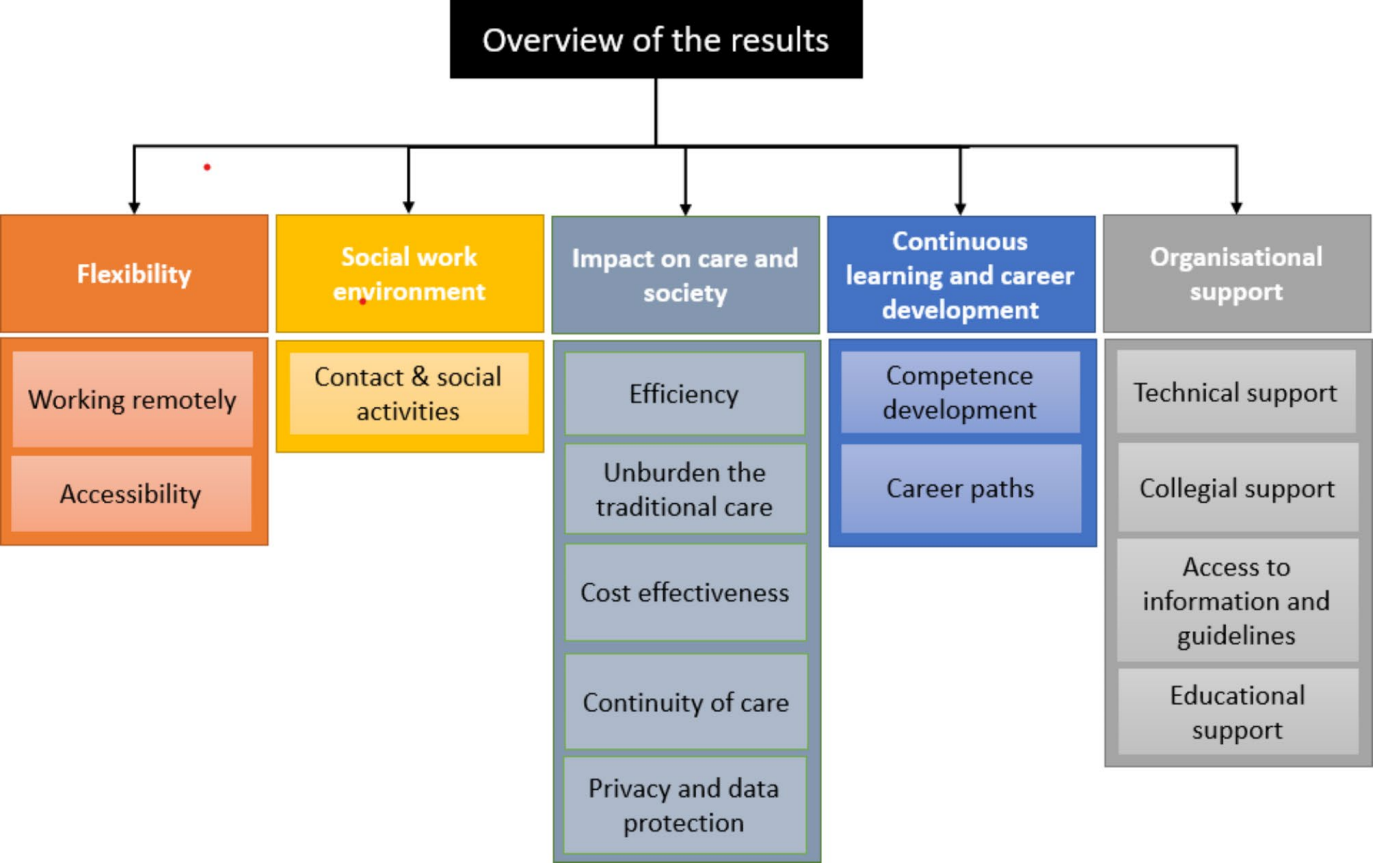
Overview of the qualitative analysis

Based on the content analysis, five categories and fourteen subcategories were identified (Fig. 1). Categories included *Flexibility, Social work environment, Impact on care and society, Continuous learning and career development*, and *Organisational support*. The categories and subcategories will be further described below. We have not observed any age or gender differences in our material, except for a difference between early- and late-career physicians in their views on career development.

### Flexibility

Participants stated that working remotely, being independent of place and time, and increasing patient access to health care are positive features of working digitally. However, they also described drawbacks with the increased flexibility.

Being able to **work remotely** was seen as one of the benefits of working in a digital context. Some participants however also missed contact with co-workers.

*“[I’ve worked] almost exclusively at home. I’ve worked from the office a few times, just to have contact with people. But I experienced… You are so isolated anyway, with their rather short breaks, and perhaps no one else has a break at the same time so it did not give as much social contact as I expected. So I almost only work from home”.* (Interview 3)

Participants were eager to work in a digital environment without being dependent on a time or place. They experienced having control over their time and being able to schedule a little freer.

*“Flexibility, that’s what they [the online healthcare provider] offer. […] If I chose to work only digitally, then I would be very, very flexible.” (Interview 10)*.

The flexibility to control when and where to work also opens up opportunities to engage parts of the workforce that could otherwise be unavailable. Clinicians who had retired or who would not consider taking on extra shifts at the clinic may choose to provide a few hours of VCs every week.

**Accessibility** was another important topic related to flexibility. Physicians believed that timely access to physicians through VCs led to less waiting time for patients and improved cost control. Even though there are some limitations with digital examinations, the participants expressed that increased accessibility improves and accelerates the diagnosis in some cases, such as examining skin lesions.

*“accessibility must be the primary benefit. There are some limitations on what can be diagnosed. […] If you are looking for skin changes then it may not need a physical examination.” (Interview 9)*.

Some participants had also experienced drawbacks of accessibility, describing situations when patients call from locations where they cannot talk (the bus, or while driving), or simply did not focus on the visit; “[…] *this young person, I could tell they were looking at something else, and I heard sounds, and it turns out they are watching a movie while talking about serious issues, I think that’s totally disrespectful.” (Interview 16).*

### Social work environment

While most participants said they had experienced loneliness in the digital environment, others did not perceive any difference between digital and physical environments in terms of social interaction. Some physicians found working in the digital environment to be calmer and less intrusive.

There were different opinions on the impact of VCs on collegial **contact and social activities**. Most of the participants expressed loneliness in working digitally all the time.

*“That’s one reason I wouldn’t work full-time with digital care. You can become isolated.* [The online healthcare provider] *offers the opportunity to sit in an office, at least in Stockholm, but even if that opportunity existed where I live, I might not go to the office anyway, since it’s so comfortable working from home. That’s why I think it works better to combine physical and digital care.” (Interview 21)*.

However, some participants did not experience any significant difference to working in primary care centers when it comes to the social aspects; *“I don’t think you could talk to your colleagues at a health care center either as you spend most of your time in your examination room. You meet patients, but you do that in the online care environment too. I don’t think it’s such a huge difference.” (Interview 22)*.

Participants who worked part-time at the online healthcare provider and part-time with another healthcare provider expressed that the social activities they had at their physical work compensated for the loneliness of the online context.

*“…otherwise I would not have done this. If I had to only work digitally, I think it would be too lonely. But not the way I work now.” (Interview 1)*.

Some of the participants expressed that even though digital work was lonely, they do not suffer from it as much as they feared and that escaping the stressful work environment of traditional healthcare compensates for some loss of social context.

*“It is more solitary work, but you can pick up the phone at any time to talk to the back office. It’s not the same thing. It is not possible to have a coffee with a colleague. But then again, I think that at the health center it was sometimes so stressful that people did not have time to eat lunch anyway.” (Interview 3)*.

Most participants described a more peaceful and less stressful work environment in the digital context, leaving room for more relaxation and space for recovery. They describe a stressful work situation in traditional healthcare, making the online care context a relief.

*“I feel that this is almost a relief, to be on my own and work in peace. […] If I worked only for an online care provider, I would miss the break room and the people. But I experience this as a relief from the heavy workload at the physical primary care center. And where we sit, in the break room, there’s so much noise. You can’t relax at all, but you also have to leave your office to take a break. But the relaxation here at home, when I’m on my own, and at the healthcare center are two very different things. I can really relax here, I can’t at the healthcare center.” (Interview 15)*.

The majority of the participants believed that most of the patients are positive towards VCs. Having satisfied patients was also described as having a positive impact on the physicians’ work environment and made them feel that their work benefits patients.

*“Yes, I think these depressed patients, of whom I have had many, are particularly successful. When you call for a follow-up visit and they feel much better and you know they could never have received this care so quickly without the online service. These patient meetings are usually very successful. Then I feel that I have really made a difference.” (Interview 5)*.

### Impact on care and society

Participants believed that VCs have a positive effect on society and the economy by providing efficient care, reducing workload in physical care centers, being cost-effective, and providing continuity of care.

Participants expressed that visiting more patients in less time has increased productivity and made the process more **efficient**.

*“It is time efficient. In physical care you have to talk to a receptionist first, and then a nurse, a triage, and then an assistant physician, maybe, and then maybe even a chief physician in the end. […] You should try to be efficient and to see as many patients as possible to do with high quality. That’s where I think these video consultations are pretty good.” (Interview 11)*.

Physicians stated that visiting patients digitally **unburdens traditional healthcare**, leaving more time for primary care centers and emergency departments to provide better care to patients who need it most; *“above all, it is possible to keep patients away from emergency rooms and to a certain extent health centers that can instead work with what they are best at.” (Interview 15)*.

Participants expressed that easy access to health care and physicians through VCs will increase **cost-effectiveness** and ultimately have a positive impact on society and the economy.

*“you can make primary care visits more cost-effective and increase the accessibility for patients. It will cost less and it will make more doctors or clinics available to patients. […] I think this is the beginning of something very good, otherwise, I wouldn’t be here. I see long-term cost savings for society and I see an increased quality of care and increased availability of care for patients.” (Interview 22)*.

**Continuity of care** is an important quality factor in healthcare, and online healthcare providers have been criticized for interrupting the continuity of primary care. Continuity can be broken down into three types, informational, relational, and management continuity [23]. The participants in this study described both informational and relational continuity in their online healthcare context.

Participants believed there is support for *informational continuity* as they have access to patient medical records through the national patient overview (NPO) [5] to confirm the patient’s history; **“…***we have access to the national patient overview so I could go in and see what it was he had been prescribed before and it corresponded to his story” (Interview 22)*.

On the other hand, not having an already existing relationship with the patient, the participants also described basing most of their assessment on the patients’ retelling of their health history or anamnesis, and if necessary, they will consult the NPO. Concerning creating *relational continuity*, the participants referred to the possibility to follow up on a patient in the digital environment as an important feature.

*“[…] and then I followed him over the summer, saw how he improved in his depression, and then he could continue his follow-up at his regular healthcare provider after summer.” (Interview 22)*.

The healthcare professionals’ availability for follow-up could however be limited due to many working only a few hours online each week.

*“In the short term [it is] great. One week, two, ahead [it works]. I think it gets more difficult later on. […] To book a follow-up visit three months ahead is not possible, because I don’t work that many hours. But for follow-ups in a couple of days, or a week or two, that’s fine.” (Interview 3)*.

Even though there is the possibility for follow-up with the same physician, sometimes patients choose to not use the opportunity.

*“But patients usually don’t choose it. Most often it was when it was a long-term follow-up, that is, we would meet again in four weeks or so.” (Interview 4)*.

Some participants also mentioned that there are limitations with continuity of care for some types of disease compared to physical care.

*“… where does the patient end up when referring? Does the patient go to the health care center or the psychologist or the hospital, or? There is no follow-up to this… That’s a limitation, of course. But on the other hand, the kind of visits I have had so far, they usually did not require follow-up.” (Interview 11)*.

*“If I think back to when I was working at a physical health care center earlier, there was a lot of follow-up of cardiovascular disease, type 2 diabetes that I have not seen in the online healthcare context.” (Interview 7)*.

**Privacy and data protection** were challenges that physicians expressed related to online VCs. A few participants were worried that using parallel communication channels to exchange patient information with colleagues could jeopardize privacy.

*“It would be that information somehow ends up in the wrong place. Pictures and things like that. Even if we delete them here at home… One hopes that it doesn’t end up somewhere where it shouldn’t be. (Interview 3)*

### Continuous learning and Career development

Some participants stated that the focus on mostly handling uncomplicated or easy cases resulted in competence development stagnation, while others saw the online care context as a learning opportunity in itself, offering opportunities for career development.

When it comes to **competence development** as a physician, some participants felt that working only with online care you run the risk of losing skills, or not gaining new skills due to not having enough variation in cases; *“the downside of working like this, is that you do not develop. Sure, you sometimes go to lectures. But you stand still in many ways as a doctor….” (Interview 5).*

This is not only related to clinical skills but also to the quite strict **career paths** available to physicians.

*“[…] in my clinical work I don’t progress much. You always improve for every patient you meet, but it is not part of a residency and that is very much what counts if you think of career steps as a physician.”(Interview 22)*.

This may be part of the reasons why some participants expressed that their work in an online context was not their core activity; “*I don’t think of digital care as a career for me, rather a job on the side.” (Interview 7).* Most of our participants worked the majority of their time in physical care and worked in parallel for the digital healthcare provider.

While some participants did not see their work as online physicians as a means to progress in their career, others saw it as a future career opportunity. They wanted to focus more on digital care and therefore saw the experience of working in a digital setting as essential.

*“I believe that this is the future, and I believe that it’s important for us to keep up with the development of society.” (Interview 19)*.

### Organizational support

All participants believed that they have sufficient and timely feedback and support.

Participants experienced that **technical support** services were provided immediately and without delay; *“…when you don’t know [how to handle the technical issues] you get technical help and you have your own contact person that you can turn to, and you can reach out to the back office so the support is very good.” (Interview 20).*

In a digital care context, eHealth systems are at the core of the care provision, and accessible technical support is essential to be able to provide online care. The participants often compared the online care context (more or less explicitly) to the technical support provided in traditional healthcare.

*“[…] as soon as a technical problem occurs, they fix it immediately. You never have to sit in the telephone queue for half a day for a technical problem.” (Interview 22).*

**Collegial support** mainly focused on supporting each other in decision-making, guidance, and consultations. Physicians mentioned that they have access to other physicians and colleagues if they needed consultation.

*“At an average primary care center, you have three colleagues, onSlack* [an online communication channel] *25 colleagues are working at the same time. There you can share cases that you think are tricky, or share frustration too. Antibiotic counseling is a classic, the patient wants antibiotics and you don’t want to prescribe them yourself because you don’t think it is necessary and then you can get support from your colleagues.” (Interview 22)*.

There may however not always be e.g. pediatrics specialists on call which was expressed as a problem by some of the participants.

Concerning **access to information and clinical guidelines**, the participants were satisfied with the situation in the online care context. Again, comparisons were often made with their previous experience from traditional care, and the respondents describe a situation where digital tools are more adapted to and easily available in the online work environment. Participants also acknowledge that it may be easier to adapt guidelines to the online care environment where the range of patient cases is limited.

*“We have good guidelines that we work on. We have adopted guidelines developed by our medical director. They are adapted to what we have to deal with at [the online healthcare provider], so all the large patient groups we handle have specific recommendations.” (Interview 22)*.

*“It is always very easy as a doctor to make sure that you are giving treatment in accordance to guidelines, unlike if you work at a traditional primary care center where you need to search in a paper folder somewhere, and then it’s not the latest version of the guideline, so you have to search in one of the big knowledge databases” (Interview 22)*.

Participants described the **educational support** in the organization as satisfactory. Training was provided as part of their onboarding, as well as continuously through e.g. case seminars and online lectures. They were familiar with the technical and non-technical aspects of the systems used. In addition, they received help when it was needed.

*“it was maybe 14 or 15 short videos, to know how the system works, how the portal works.” (Interview 6)*.

Apart from the training provided by the online care provider, none of the participants had received any education regarding VCs elsewhere (e.g. in their undergraduate education to become physicians). Yet, they felt that it was relatively easy to adapt to online care; *“[…] but all that you have done before at the primary care center, it means that you have a foundation to stand on even in the video consultation.” (Interview 3)*.

## Discussion

We were able to identify five main themes, each with several subgroups (Fig. 1). What could most clearly be interpreted as an advantage or benefit of working with online VCs was flexibility due to teleworking, and the freedom to influence one’s schedule. Working from home was perceived to mean both fewer disruptions and fewer conflicts (compared to working at a physical, traditional clinic), and thus had a positive effect on the perceived work environment. The participants also felt supported by the organization, through collegial forums and contact channels to discuss patient cases, as well as through easy access to technical problem-solving. At the same time, fears and risks related to data security were expressed, which of course requires both secure working methods and routines, as well as compliance with these. The importance of patient privacy together with cybersecurity was also addressed by Shah and colleagues [24].

### Strengths and limitations

This study is one of the first studies focusing exclusively on the experiences of physicians working for an online healthcare provider. It gives important insights into their experiences of the online work environment and how it differs from the work environment at a physical healthcare center. Saturation of data was not discussed during the interviews, rather all volunteering participants were included. Yet, study participants represented a wide range of ages, years of work experience, and proportion of work dedicated to online care.

Participant checking of the transcribed interviews or analysis was not done. These are weaknesses to the rigor of the method that needs to be taken into consideration. The physicians relevant to our study however constitute a population with a high workload and limited availability, and we were reluctant to add more burden to their participation in the study. During the qualitative analysis, we chose not to have two or more coders per interview, which could be considered a weakness of the study. We however had frequent discussions of the codes to reach a consensus, and all researchers read all interview transcripts.

### Continuity of care

Regarding perceived continuity, the physicians stated that there was an opportunity to ensure both information and the patient-physician relationship, but that many patients chose not to be treated by the same physician in favor of faster help. In a recent study among Norwegian GPs, there was a stark contrast between the perceived quality of VCs when the patient was known and when the patient and GP did not know each other [6]. When GPs knew the patients well beforehand, VCs were considered equally or better suited than face-to-face consultations in 57% (1011/1785) of cases, as opposed to 32% (87/274) when the patient was previously unknown [6]. Follow-up consultations were also rated as suitable by 61% of the respondents, compared to only 35% for new problems [6]. The participants in our study did not express similar concerns, but we need to keep in mind the difference in context. In a recent report from the Swedish Agency for Health and Care Services Analysis comparing Swedish healthcare to ten other countries, Sweden stands out, especially with regard to relational continuity. In Sweden, only 35% of respondents had a regular healthcare provider (physician or nurse), compared to between 80 and 98% in the other countries [25]. Perhaps this is part of the explanation as to why this is not seen as more of a problem in our study, few physicians experience relational continuity in physical care making the lack thereof in VCs less tangible. In our study, informational continuity in the form of access to patient data and treatment guidelines was highlighted and was perceived as an important prerequisite for being able to offer patient-safe care.

### Education and career development

The physicians in our study felt that they had received sufficient training from the employer in question to be able to provide good quality online care. At the same time, career opportunities were considered limited, as the work could not be included in any formal part of their clinical education, i.e. residency (ST in Swedish). The work was thus seen as more of a side job; only two of our participants worked full-time in digital care, and another two worked part-time in digital care without having other clinical work. The majority of our participants were employed by a physical healthcare provider and in parallel worked between 3 and 20 h/week for the digital healthcare provider. On the other hand, their engagement in digital care was felt to provide an opportunity to develop digital skills relevant to future health care.

### Work environment in a digital care context

Karasek and Theorell identified job demands and job control as key factors influencing the work environment [26] [27]. Job demands include time pressures and role conflicts, whereas job control relates to an employee’s opportunity to control their work situation [26]. Jobs with high demands and low control (“high strain jobs”) carry a high risk of developing adverse psychological symptoms such as anxiety and depression, but also cardiovascular disease [26]. In our study, several of the participants describe their work for a digital care provider almost as an escape or relief from their high-demand work in physical healthcare. In the digital care context, they experienced high levels of control in scheduling their work and in working from home and did not have to deal with the added demands of being in a busy workplace. Although time pressure is high also in the digital context, the participants did not experience the same level of stress as when patients are sitting in the waiting room.

The Karasek and Theorell model was later extended by Johnson and Hall [28] who added the dimension of “support” at the workplace. The support dimension can moderate the negative impact of high strain so that employees can work with high demands without experiencing stress, negative mental health impact, and job strain. The model predicts that work situations characterized by high demands, low control, and low social support are the most harmful to workers’ well-being [28]. In our study, the participants described different types of support; collegial support through online forums, and technical support that was fast and efficient (often contrasted with dysfunctional support in physical care). Poor usability of eHealth systems is a well-known problem, in Sweden [29] and internationally [30–32]. Our participants’ frustration with the eHealth systems and technical support in physical care, was described as part of the reasons for working for an online healthcare provider. Stress and clinician burnout related to health information technology has been identified in several studies [33, 34] and remains a growing issue as the digitalization of healthcare continues. In a digital care context, eHealth systems are at the core of the care provision, and high usability is essential to be able to provide online care. As VCs become a more integrated part of all healthcare, focus on usability and technical support will need to increase to ensure high-quality care and reduce the risk of clinician burnout.

Patients’ appreciation of the high level of availability of the digital services, were perceived as bringing positive energy into the digital consultation and the work environment. The physicians experience of patient satisfaction with VCs corresponds to recent studies of patients’ experiences of of online care [11, 35]. In addition, several different patient cases were described as clinically benefitting from the remote solution, e.g. due to the long distance to the physical care unit and everyday logistics for parents of younger children. This easiness and flexibility was also shown by Björndell & Premberg in a study of primary physicians who had digital possibilities within the ordinary primary health care provider [36]. In our study, we could also see a tension between the benefits brought by increased flexibility and a frustration with patients taking this flexibility too far by e.g. connecting while driving or on a crowded bus.

The majority otherwise felt that physical care could be relieved through digital handling of simpler cases in a more cost-effective way. In a recent study of patient and provider satisfaction with VCs, patients with easier chief complaints appreciated the VC more, whereas patients who assess their situation as complex seem to prefer face-to-face consultations [37]. Determining whether VCs can actually ease the burden on traditional primary care would require further studies, i.e. quantitative data are needed to be able to de facto show a relieving effect, as the total case mix in physical primary care is greater, not least with the management of chronic conditions. In addition, it would be relevant from an educational perspective, and to build up and maintain a broad clinical competence, to be able to handle all kinds of cases as a doctor and not just the simple ones in a digital care environment (which Fernemark et al. also concluded in a 2020 publication [38]). This limitation of patient cases that are suitable for VCs was also highlighted by our participants.

In this study, we only interviewed physicians who worked fully (few individuals) or partially (a majority) for a private online healthcare provider. We therefore cannot compare their experiences to physicians who work with telemedicine or VCs as an integrated part of their work at a traditional primary care center. However, all of the participants had experience of working in traditional, physical and public health care and reflected on the differences between the contexts. Several recent Swedish studies also focus on the experiences of clinicians working with VCs integrated in traditional care [36, 38], and our study therefore provides a complement with its specific focus on this context. We also believe that although the contexts differ, many challenges are common, and there are lessons to be learned between contexts. Yet, further research is required into the differences between introducing VCs in a traditional healthcare organization and in an exclusively online organization.

The corona pandemic year 2020 has led to a massive increase in digital healthcare services nationally and internationally [5, 6, 39]. Thus, the importance and the need for telemedicine solutions have been highlighted due to the pandemic. This has led to telemedicine solutions being integrated to a greater extent in everyday clinical work also in traditionally physical healthcare contexts. In addition, the online private healthcare providers in Sweden have opened their own or taken over physical primary care centers, further increasing the integration between physical and digital care. What impact this integration of online and physical care will have on physicians work environment remains to be explored. Many of the benefits described in this study (flexibility, control, support) may be dependent on the fully digital care context. When VCs are introduced into a traditional healthcare organization, the impact on work environment may be different.

## Conclusions

VCs seem to be an appreciated phenomenon, mainly because physicians feel that the context offers flexibility in their everyday work. The high availability seems to be appreciated by the patients, which in turn is perceived to have a positive impact on the work environment of the physicians. Potential positive system effects, such as relief of physical care and cost-effectiveness, need to be investigated in more detail in quantitative studies.

The pandemic year 2020 has proven, nationally and internationally, to lead to a significant increase in the implementation and integration of VCs, and other remote services, in traditional care systems. However, the need for educational efforts in both technology and context remains, as well as integration into physicians’ formal education, theoretical and clinical, at different levels. Usability of telemedicine and eHealth systems is of essence, and needs additional focus when in the future VCs become an integrated part of all healthcare provision. Ensuring high-quality eHealth systems and technical support will be essential in ensuring continued digitalization of healthcare without increasing the risk of clinical burnout. These should be important prerequisites for supporting physicians in both adoption of and adaptation to the digital care environment.

### Electronic supplementary material

Below is the link to the electronic supplementary material.

Supplementary Material 1

## Data Availability

The datasets generated and analysed during the current study are not publicly available since the qualitative data collected cannot be shared without risking the study participants privacy. For inquiries regarding the dataset, please contact either the first author of this study (maria.hagglund@kbh.uu.se) or Uppsala University’s data protection officer (dataskyddsombud@uu.se).

## List of abbreviations

ICT: Informatin and Communication Technology
NPO: National Patient Overview
VC: Video Consultation
