# Supplementary material for: Escape to the future – a qualitative study of physicians’ views on the work environment, education, and support in a digital context
Source: BMC Med Inform Decis Mak. 2023 Oct 19;23:231. doi: 10.1186/s12911-023-02337-7 (PMC10588019; doi:10.1186/s12911-023-02337-7)
Supplement: Supplementary file 1 — Supplementary Material 1 [file 12911_2023_2337_MOESM1_ESM.pdf]

Interview guide clinicians

Short introduction to the project and questions.

BACKGROUND INFORMATION

1. How long have you worked as a physician in a digital context?
2. Do you work exclusively digital/online?
3. Why did you start working for this online healthcare provider?
4. Have you ever worked for any other online healthcare provider?
5. From where do you work?
6. What do you think is signifies an online video consultation? How is the digital context different from the physical?

ABOUT EDUCATION AND COMPETENCIES

7. Do you feel like you medical education prepared you for working online/with online video consultations?
8. Have you had any education or training for working with online video consultations?
9. Is there anything you would like more training/education in?

EXPERIENCE OF THE ONLINE VIDEO CONSULTATION

10. How do you think the patient experiences the digital context?
11. Have you noticed any difference in patient population using the online video consultations compared to traditional care, i.e. do you experience that *other* patients seek online care than physical?
12. What are the positive effects or opportunities of online video consultations?
13. Tell me about a successful online video consultation.
14. What challenges, difficulties, or risks do you think there are with online video consultations?
15. Tell me about a situation where an online video consultation was difficult.
16. How does the context (online or physical consultation) affect communication (the patient-physician relationship/therapeutic alliance)?
17. What factors do have you experienced as affecting the online video consultation? (e.g. technology, internet connection)

a. *Do you experience frequent problems with the service?*
b. *Do you experience that patients have problems using the service?*

ON WORK ENVIRONMENT / QUALITY OF CARE / PATIENT SAFETY

18. How do you consider the possibility for continuity, follow-up in an online context?

19. Does the support you have in your clinical work (access to records, senior consultants, guidelines) work in the online context compared to a physical work environment?

20. Do you have access to the information you need in your online work?

21. What are your thoughts on the clinical decision making in an online environment? *[do you feel that you have/can have access to all the information you need? Do you base your decisions on different information in a digital vs physical context? How is shared decision making affected?]*

22. Do you experience that you have the opportunity for collegial support in the online work environment, e.g. informal discussions of patient cases?

23. How do you experience career development/opportunities and professional growth in digital care today?

OTHERS' OPINIONS ON DIGITAL CARE

24. What attitudes towards digital care have you met?

    [What do you think is the reason for these attitudes? How do you view these issues?]

    [IF no negative opinions have been experienced] – In the public debate, certain issues – e.g. reimbursement models, patient safety, antibiotic prescriptions – have been raised. What are your thoughts on these issues?]

25. Do you tell people that you work for an online healthcare provider? What reactions do you get?

CLOSING QUESTIONS

26. What do you miss in the online video consultation?
    a. *Do you have any improvement ideas?*

27. Is there anything we have forgotten to ask? Anything you would like to add?
